# Supplementary material for: Patterns of Intron Gain and Loss in Fungi
Source: PLoS Biol. 2004 Nov 30;2(12):e422. doi: 10.1371/journal.pbio.0020422 (PMC532390; doi:10.1371/journal.pbio.0020422)
Supplement: Table S1 — Also available at http://genes.mit.edu/NielsenEtAl/. (4.3 MB ZIP). [file pbio.0020422.st001.zip › NielsenEtAl/html/1376.html]

AN3752.1.NCU02211.1.MG01976.1.FG05780.1


```
 CLUSTAL W (1.82) Multiple Sequence Alignments - Introns Inserted


Sequence 1: NCU02211.1	725 aa
Sequence 2: MG01976.1	766 aa
Sequence 3: FG05780.1	707 aa
Sequence 4: AN3752.1	741 aa
Alignment Length: 802 aa
Number Identitical Residues: 208 aa
Alignment Score (without introns) 11744


MG01976.1 	MMLHEPAMANHLASMKRTDSPPGLTGSKSSKSSSFHSRN--SDDSSVLADVSHFEEIGLD
NCU02211.1	MMLYERSVH----DLPGPSSPPGLTHSKSSKSSSLNSVP--SDDDSVLADASHFEDIGLQ
FG05780.1 	-MIRTLDIG---SALPGPDSPPGMTTSKSSKTSSFQSYD--SDDASVMADIGHFEEIGLD
AN3752.1  	-------MA---AVLTAPNSPPELSGSKSSKSSSYRSSSRMSSPDGVFTDIGNFEEIGLE
          	       :    : :  ..*** :: *****:** .*    *.  .*::* .:**:***:

MG01976.1 	DVATHLER--EVPVKTAPNPFSASYPTDLRAASRTKSAPRLPAPRDLGKTRPLREITTGT
NCU02211.1	DESHVDVRGLTKNLKPSPMLYSSNPSSELRVPSKSSRSSRSPS-----KTRQKRDVSSTG
FG05780.1 	DD--------SVTLK-SPSKVDVRPSLPKAHSSRSLVAGKTPS-----------------
AN3752.1  	DE--------SAPYLESSVSHGRAPGVARSSTARILAKSPVST-----------------
          	*               :.   .         .::       .:                 

MG01976.1 	KTRPKFPPLHTTDLRTINVRSTNLNTLYPAPMTSPIRSKSLGPRNAAILPSRRRRSPSPN
NCU02211.1	KVRPTLPNIQS-HLRPVNGRVASLG-LTPELHTAPLPMRSLGNRSP---SSTRQRSISPN
FG05780.1 	KARPPFPTLET-NVYTSNPRSTNLSALTEPLSAT--RPKTLTSPSSASLTFNRRRSPSPA
AN3752.1  	-TRDLLSAAPRKPYASQGRPGPRLMTASRDLQTPRPSSKTRRNRSASPLRPSQSPGIASS
          	 .*  :.    .   . .   . * :      :.    ::    ..:     :  . :. 

MG01976.1 	LSMVSRDPSIS--MIRPRRSSWQATHERKSSLELERECDEDDDDDIPDYLILDNVPISPR
NCU02211.1	LQTSFP--------MKPRRSSWQSSRERKTARQLERECDEDDGDDIPDGLVLDNVPISPR
FG05780.1 	SSLNPRDPREPSFAAKPRRSSWQTARERKSFTDLERECDEDDGDDIPDGMFLDNVPLSPR
AN3752.1  	PSSQTLSPSAVNARPLNRKPSWQ--RSRKSLKELEAEYHDSD-DELPEDASLWNIPISPR
          	 .    ..   .     *:.***  :.**:  :** * .:.* *::*:   * *:*:***

MG01976.1 	PQSERSRSQASSKANSPDRGPKE-RVRSIGNGTPAVAMAHGSLRSPTWKTDGALASPPTP
NCU02211.1	PPSERTISQPSSKAPSPERAPPEKRVRSVGNGTPPVAVAQGSLKSPTWKSDTAVPTR---
FG05780.1 	PMQERTPSRPASTSPSPNR-SKE-RVRSVGNGTPAVAQASGSLRSPTWKVD---------
AN3752.1  	PVEDRAPSRDHSPNRSPGPQPFP-LSHSVSEAAVASGPPRRSPATSRANRQMARSSS---
          	* .:*: *:  *   **   .     :*:.:.: . . .  *  :.  : : : .:    

MG01976.1 	HGHNLISTPPSGTVSPMK-TRTKSWTVALAELNAEAKELTEKLEEHADELQQKSAQRSS-
NCU02211.1	------ASIASTIVTPLELARSKSWSAALAELNSDAKELTEKLEEHAEELEAK-GVRSS-
FG05780.1 	-----------APRSPAQ-TRAHSWNAALAELNAEAKTLTEKLEEHADELVEKHIKRPAN
AN3752.1  	------AGPERGQISPRN-PRLYSYNSMMSDLSEEAKNLTQALEFHADENDRKRGDSLRS
          	      :       :* : .*  *:.  :::*. :** **: ** **:*   *      .

MG01976.1 	-TGSMPTNRPRSADSNDTKTRHKSALPELPPLRRSNIMIDPLPISKEKEAVLSRTRPSWL
NCU02211.1	-TPSVRRN-------SDGKPRVKSGLPELPPLRKSNVMIDPLPPSKEKEAVLSRTRPSWL
FG05780.1 	QRPNTWNASRRSADMYEKKQRVRS-TPELPPLRKTNIMIDPLPVSKEKEAVLSRTRPSWL
AN3752.1  	GNSSLRSS-------DESKRDSKS-PIELPPVQKSNIMIDPLPISKEKEKVLSRTRPSWL
          	   .            : *   :*   ****::::*:****** ***** **********

MG01976.1 	PPKDPAEERRHLKEYQKMMAKSAEADRRRETAKKLKSECKDVAADTLMQVWEFDVLPRWN
NCU02211.1	PPKDPAEERRHLKEYQRMMAQSLEAERRREATKKARIEGRDTRADNLMHIWEENIIPRWD
FG05780.1 	PPKDPAEERRHLKEYQKMMAASVKAEERREASRRAKVEGRDSTADNLMQIWERDVIPRWN
AN3752.1  	PPKDQKEERRHLREYKKMMEQSREADKRKAAQAASAKCERDNTRDTLQQIWDDYVYPNWT
          	****  ******:**::**  * :*:.*: :        :*   *.* ::*:  : *.* 

MG01976.1 	EAIRERRTRELWWRGVSPRSRGAVWTRAIGNELGLTETSYRAALRRAREAEEREAAGKST
NCU02211.1	EAIRERRTRELWWRGIAPRSRGAVWSKAIGNGLALSDASFRAALKRANDAQKRVNDGQGS
FG05780.1 	EAIRERRTRELWWKGIAPRSRGVIWGRAIGNELGLSETSFKAALARAQEIETRVKDDKGD
AN3752.1  	RAIGEPRTRELWWRGIPSRIRGQTWNRAIGNELALSDESYNKALKRAKDARAK-SEGDSG
          	.** * *******:*:..* **  * :**** *.*:: *:. ** **.: . :   ... 

MG01976.1 	SADQKYAGWMRAIRRDVEKATWIDLRIFQKGGPLHQSLVDILSAYAMYRSDIGYVAGCN0
NCU02211.1	TDDSYAAAWFDDIKKDVEEYTFPDLRIFQTGGPLHQSLVDVLSAYCMYRSDIGYVPGCN0
FG05780.1 	AEDARRAKWFQEIRKDASHNTWQDLRIFDVEGPLHQSLVDVLSAYAMYRSDIGYVRGCN0
AN3752.1  	ESNKKMMQWFDAIQADVSK-AFPELNLFQEGGPLRETLVDVLEAYAMYRSDVGYLTGLH0
          	  :     *:  *: *... :: :*.:*:  ***:::***:*.**.*****:**: * : 

MG01976.1 	TMAALLLLNLPSATESFIALANVLNRPLPLSFYAADPGAKATAYNLILQTLAHKSPALHN
NCU02211.1	TIAAILLLNLPSATDAFIALANVLNRSLPLSFHTCDRTAMSSAYNLLLQTLQHKSPSLHE
FG05780.1 	TIAALLLLNLPDVPSAFVALANVLNRPLPLSFYTGDPGAQASAFNLVMQTLSLKSAPLHA
AN3752.1  	TIAALLVLQFPTPSSAFCAMANALNRPLPVAFMTMDHGAIGRTFSLASATLRYKFPRLAT
          	*:**:*:*::*  ..:* *:**.***.**::* : *  * . ::.*   **  * . *  

MG01976.1 	HITN-LPDHDPELYMCDLFTGLFTSHLPLDAAARLWDVYVFEGDKLLIRAAVALMAENEM
NCU02211.1	HLIK-LPDHNPDLYLGDIFTGLFTRHLALDEATRLWDVYVFEGDAVLVRAGIALLLQKGM
FG05780.1 	HLTKKIPTAELEQSLSGILTAVFTQHLAIDEAARLWDVYVFEGDALLIRAAVALLLSREM
AN3752.1  	HLYETLRLSDEEIFES-MFRSLLTNGLDLERLSRVWDCWVFEGDRIFIRAAVAILGCLQT
          	*: :.:   : :     :: .::*  * ::  :*:** :***** :::**.:*::     

MG01976.1 	SLLG-TASASEVHTVLQGRSCTTASSSPGSDKHAKSQR--------------------VV
NCU02211.1	SLMG-TKTIHEVRAIMNN----------SSDGVLRSAP--------------------IL
FG05780.1 	TLLG-AKTAEEVKTILNE-----------RNAKVSSVR--------------------VA
AN3752.1  	QLFGFTEPDDQSRLAVKNILAWGPHDIGTKPKERRSAPAAPIAGFAGGLIGAAAGHYWIL
          	 *:* : .  : :  ::   .  . .         *  ::. :. :..  .:::.   : 

MG01976.1 	GRAGEEDAWINAVRSAGKV----
NCU02211.1	RSTGEEDRWMRAVREAGKAQA--
FG05780.1 	GEVGAEDKFMTSVREAGKA----
AN3752.1  	TSAGDEDGFISEMREAGKVQPRA
          	  .* ** ::  :*.***... :
```
